# Love at second sight: Sequential dependence of facial attractiveness in an on-line dating paradigm

**DOI:** 10.1038/srep22740

**Published:** 2016-03-17

**Authors:** Jessica Taubert, Erik Van der Burg, David Alais

**Affiliations:** 1 The School of Psychology, The University of Sydney, NSW 2006, Australia; 2 Experimental and Applied Psychology, Vrije Universiteit Amsterdam, the Netherlands

## Abstract

Millions of people use online dating sites each day, scanning through streams of face images in search of an attractive mate. Face images, like most visual stimuli, undergo processes whereby the current percept is altered by exposure to previous visual input. Recent studies using rapid sequences of faces have found that perception of face identity is biased towards recently seen faces, promoting identity-invariance over time, and this has been extended to perceived face attractiveness. In this paper we adapt the rapid sequence task to ask a question about mate selection pertinent in the digital age. We designed a binary task mimicking the selection interface currently popular in online dating websites in which observers typically make binary decisions (attractive or unattractive) about each face in a sequence of unfamiliar faces. Our findings show that binary attractiveness decisions are not independent: we are more likely to rate a face as attractive when the preceding face was attractive than when it was unattractive.

Social dynamics in the contemporary world are rapidly changing in response to online technologies. For many, online profiles on sites such as Facebook and Instagram contribute as much to their social lives as their real-world interactions. This is especially true for dating practices, which have been revolutionized by the internet and translated into huge business with millions of users each day logging on to online dating sites in search of potential mates.

While online dating is popular, and is certainly an efficient (and anonymous) way to sort through potential mates from the comfort of one’s own home, it may not be quite as reliable as it seems given the recent evidence for sequential dependencies when judging rapid sequences of faces^1^,^2^. In this paper we explored the social phenomenon of online dating using a simple binary task that mimics the procedure used by immensely popular online dating sites such as e-Harmony, OK Cupid, Tinder, etc. Two experiments are described that explore the impact of serial dependence on face attractiveness perception in a simplified, real-world context. The experiments employ a rapid adaptation paradigm that has emerged recently in the perceptual sciences whereby subjects are asked to make quick judgements about a rapid sequence of randomly varying stimuli. This method has been used to show robust inter-trial adaptation in judgements of orientation^1^ and numerosity^3^ in vision, frequency in audition^4^ and perceptual synchrony between audiovisual signals^5^,^6^. With regard to face stimuli, it has been reported that face identity shows inter-trial adaptation dependencies^2^, as does face attractiveness^7^,^8^,^9^.

The observation of rapid sequential dependencies in face perception raises an interesting question about the way we judge the attractiveness of unfamiliar people who post profile pictures on online dating websites. In this context, users make sequential, dichotomous decisions about whether a face is attractive or not based on a brief glimpse of a profile picture. It is not clear whether sequential dependencies are robust enough to occur when attractiveness ratings are simplified to a simple dichotomy of attractive or not, as favoured by many online dating sites. Moreover, previous studies^7^,^8^,^9^ have used laboratory stimuli, controlling for low-level visual properties for the purposes of drawing inferences about the visual system. An open question is whether these sequential effects still influence our behaviour when real profile pictures are used without the benefit of low-level control.

The current experiments will test this using a simple dichotomous task, and will do so with the well-controlled face stimuli typically used in laboratory studies of face perception replaced by non-standardised real-world profile pictures downloaded from the public domain. Our findings show that a binary attractiveness rating of a given face is strongly biased by the face seen immediately prior. In short, if the face you just saw was attractive, you are more likely to judge the current one as attractive, and vice versa.

## Results

### Experiment 1

**Figure 1:**
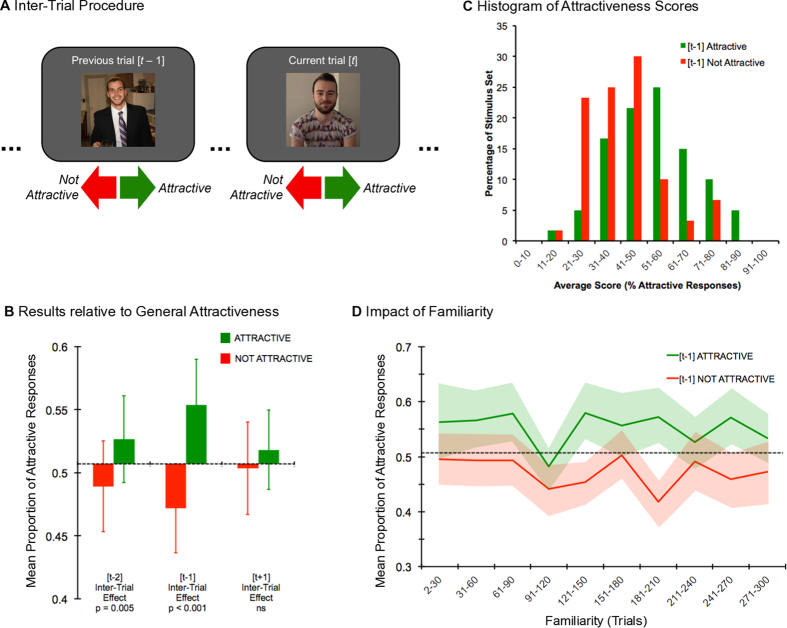
(**A**) General procedure (arrows and labels are for illustrative purposes only and were not visible during the experiment). Stimuli depicted are examples of photographs taken of men who consented to have their images reproduced for scientific communication. 300 faces were briefly presented in a random sequence and participants made a binary attractiveness judgement about each one: attractive or not attractive. (**B**) Bar graph of main results, averaged across subjects (N = 16; error bars = ±1SEM). Horizontal dashed line indicates general attractiveness (mean attractiveness score for all faces averaged across all subjects). In the centre is the [t − 1] inter-trial effect, an assimilative effect whereby the attractiveness of a current face is higher when preceded by an attractive face, and lower when preceded by an unattractive face. On the left is the [t − 2] effect, showing a weaker but still significant assimilative effect (t_15_ = 3.27, p* *< 0.005, paired two-tail t-test). As a means of control, we calculated the [t + 1] effect. As predicted, this produced a null result as the attractiveness of an unseen future face, whether attractive or unattractive, should not alter the attractiveness of the currently viewed face (t_15_ = 1.48, p = 0.16, paired two-tail t-test). (**C**) The distribution of responses (% of trials where the response was ‘attractive’) across the stimulus set (N = 60) as a function of previous-trial attractiveness. Green bars reflect the distribution of scores when the preceding trial presented an attractive stimulus (with an average score greater than a subject’s grand mean); red bars reflect the distribution of scores when the preceding trials presented an unattractive stimulus (with an average score less than a subject’s grand mean). (**D**) Time course of the [t − 1] inter-trial effect plotted over 10 intervals of 30 trials showing the effect of the preceding face’s attractiveness on the current trial was consistent across the entire trial block. The dashed horizontal line indicates general attractiveness as in panel (**B)**.

Sixteen female undergraduate students from the University of Sydney served as participants, with half the group (n = 8) judging the attractiveness of Set A faces and the other half the Set B faces (the complementary sets to be used later). A trial started with a face drawn randomly from the set of 30. In an unspeeded binary task participants judged a face as attractive or not (see Fig. 1A) and the next face followed immediately.

For each subject, we calculated the mean of the 10 attractiveness judgements for each face and the overall mean attractiveness for the whole set of faces. Faces with mean attractiveness less than the overall mean were categorised as not attractive, or as attractive when exceeding the overall mean. We then analysed each subject’s sequence of attractiveness judgements, binning them into two groups based on whether a given face was preceded by an attractive or unattractive face. Comparing these groups allowed us to test whether face attractiveness on the current trial [*t*] was contingent upon the previous [t − 1] trial’s face being attractive. A two-tailed paired *t*-test yielded a significant inter-trial effect (*t*_15_ = 4.13, *p *< 0.001; see Fig. 1B), indicating faces were judged more attractive when preceded by an attractive rather than unattractive face. Figure 1B also illustrates that while this simple procedure elicited a significant [*t* *− *2] inter-trial effect, there was no evidence of a corresponding [*t* + 1] effect, which aligns these data with previous observations of serial dependence in face perception^2^,^7^,^8^,^9^. We also calculated the degree of autocorrelation in the random sequences of trials presented in Experiment 1. The group mean data revealed that none of the non-zero lags were significantly different from 0. This result converges with the [t + 1] analysis to suggest that the sequence of trials alone could not account for the [t − 1] effect reported in Experiment 1.

Following the experiment, the eight subjects in each group selected the 15 most attractive faces from the set of 30 they had not seen during the experiment (either Set A or Set B). Thus, each image received an independent attractiveness rating given by the number of times subjects from the other group selected it as attractive. The ratings clustered into two groups around the median, with ratings >4 considered attractive and <4 unattractive. We then compared mean attractiveness on trials preceded by an attractive face with mean attractiveness on trials preceded by an unattractive face using a paired *t*-test (two-tailed) and found the same inter-trial effect based on these independent ratings (*t*_15_ = 2.87, *p* = 0.01).

An interesting question is whether all face images were dependent on the attractiveness of the face on the preceding trial or not. Figure 1C illustrates the distribution of the profile pictures as a function of the attractiveness score. Visual inspection of this figure confirms the notion that the whole distribution (and not just a part) shifts to the left (i.e., less attractive) when the preceding face is not attractive, and to the right when the preceding face is rated as attractive.

In the experiment, each of the 30 faces was presented 10 times in a random order. These repetitions were necessary as we averaged the 10 responses to obtain a reliable estimate of each face’s attractiveness. However, it is possible that by repeating the faces our participants became familiar with them, raising the possibility that familiarity may modulate the inter-trial attractiveness effect. We tested if there was any effect of familiarity by dividing the 300 trials of the main experiment into 10 consecutive blocks of 30 trials and calculating the attractiveness effect within each block. The results are plotted in Fig. 1D and show a consistently higher attractiveness score when a current face is preceded by an attractive face compared to an unattractive face. To test the significance of this difference we conducted a 2 × 10 repeated-measures ANOVA which revealed a main effect of inter-trial adaptation ([*t *− 1]_attractive_ > [*t *− 1]_not attractive_; F_1,15_ = 17.21, p* *< 0.01). There was no evidence of a main effect of familiarity (*F*_9,135_ = 0.90, *p* = 0.53) and importantly there was no interaction between inter-trial adaptation and familiarity (*F*_9,135_ = 0.90, *p* = 0.53). These results imply that increasing familiarity with the stimulus set throughout the experiment did not affect the inter-trial attractiveness effect.

Our results show that face attractiveness judgements are strongly influenced by the attractiveness of a preceding face, regardless of whether attractiveness is rated by the same observer or an independent rater. This inter-trial behaviour is intriguing in itself, as it clearly demonstrates the choices of millions of online daters are affected by the most recently seen face. However, it is interesting to know if this behaviour is driven by a low-level perceptual mechanism (e.g., sensory adaptation) or a higher-order influence such as response bias. To investigate this we ran a similar experiment randomly interleaving upright with inverted stimuli.

### Experiment 2

Another way to examine whether the [*t *− 1] effect is cognitive or perceptual is to conduct an experiment with stimuli alternating randomly between upright and inverted orientation. If the [*t *− 1] effect has a perceptual locus, the effect should be replicated between consecutive trials where stimulus orientation is held constant but is less likely to occur when the face orientation switches between two consecutive trials, because turning faces upside down has been shown to disrupt almost all perceptual processes underlying face perception, including those that allow for face detection and feature integration^10^. By contrast, a bias to repeat responses entrained by the speed of the task or when presented with a difficult-to-rate stimulus should occur regardless of image inversions.

**Figure 2:**
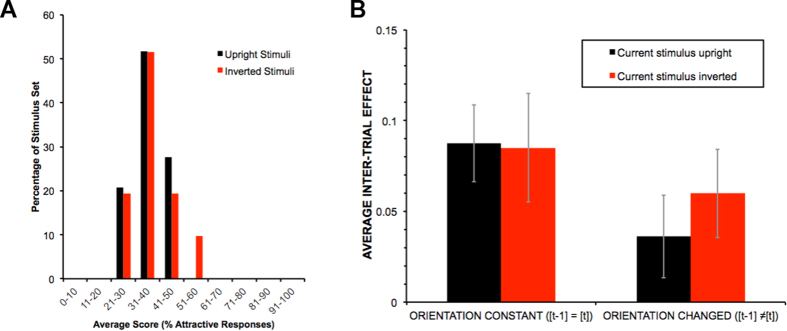
(**A**) The distribution of responses across the stimulus set (black bars when the stimuli were upright, red bars when the stimuli were inverted). (**B**) Results of Experiment 2: the effect of inter-trial orientation. The inter-trial attractiveness effect shown for all four orientation conditions. The two left-hand columns show congruent inter-trial face orientation and the two right-hand columns show incongruent inter-trial orientation. The data are group-averaged (N = 16) inter-trial attractiveness differences and error bars illustrate ±1 SEM.

An independent sample of 16 undergraduate female students was recruited and the same set of 60 faces used in Experiment 1 was used in Experiment 2 and most procedural details were unchanged. The 60 faces were judged 10 times each in a pseudorandom order. For comparison, the distribution of responses (% attractive) are provided in Fig. 2A separately for upright and inverted stimuli. Although these distributions are similar, there was no evidence of a correlation between upright and inverted attractiveness scores (N = 60, *p* = 0.56).

The results of the interleaved orientation experiment are plotted in Fig. 2B. We compared consecutive trials where face orientation was congruent (both upright or both inverted) with consecutive trials where orientation was incongruent (upright then inverted, or vice versa). First, in case there were attractiveness differences between face orientations, we binned faces as “attractive” or “unattractive” separately for upright and inverted faces. This was done as in Experiment 1: if the average attractiveness of the [*t *− 1] face was less attractive than the average of the current face, it was binned as “unattractive” and otherwise as “attractive”. We then computed the difference between current trial attractiveness scores when the [*t *− 1] trial was more attractive than the subject’s mean compared to when the [*t *− 1] trial was less attractive than the subject’s mean to get the [*t *− 1] effect separately for congruent and incongruent orientation trial pairs.

We ran a 2 × 2 repeated-measures ANOVA on the data with current [*t*] stimulus (upright vs. inverted) and congruency (congruent vs. incongruent) as within subject variables. The analysis yielded a significant congruency effect (*F*_1,15_ = 5.33, *p* = 0.03, η_p_^2^ = 0.26) indicating a stronger inter-trial effect when two consecutive faces were congruent in orientation (*Mean* = 0.09, *sem* = 0.02) than when they were incongruent (*Mean* = 0.04, *sem* = 0.02). The interaction between congruency and current stimulus was not significant (*F*_1,15_ = 0.63, *p* = 0.44, η_p_^2^ = 0.04). The main effect of current stimulus was also not significant (*F*_1,15_ = 0.15, *p* = 0.70, η_p_^2^ = 0.01).

The results of Experiment 2 are consistent with the inter-trial attractiveness effect containing a perceptual component because the effect was dependent on relative orientation between trials while the response task was always the same. However, it is likely the perceptual effect does not explain all the data, as the inter-trial effect was in general greater than zero, suggesting a role for response bias, perhaps driven by the subset of hard-to-classify faces that tended to elicit a repeat of the previous response. Importantly, however, the origin of the effect does not change our conclusion, now demonstrated in two experiments, that binary attractiveness judgements for rapid sequences of faces are reliably influenced by the recent past, whether by the previous stimulus or by the previous response to that stimulus.

## Discussion

From an evolutionary perspective, attractiveness is a key social characteristic that determines how approachable or desirable we are. Perceived attractiveness is determined not only by our own attributes but by the attractiveness of people around us^11^,^12^. These dynamics, however, need to be revisited because the way we interact with others is changing. Here we have extended work using rapid face sequences^7^,^8^,^9^ by adapting the paradigm to simulate the simple dichotomous decisions made increasingly popular in online mate-selection. Our results show that assimilative face effects are both quickly acquired and robust enough that even dichotomous attractiveness decisions are biased towards the previous trial. For people sorting through faces in search of an attractive mate, it is a case of love at second sight: their final choice of desirable mate is likely to be one face too late.

## Methods

### Participants

Across Experiments 1 and 2 we collected data from 32 female undergraduate students enrolled in third-year Psychology at the University of Sydney (NSW, Australia). All aspects of the data collection and analysis were carried out in accordance with guidelines approved by the Human Research Ethics Committee of the University of Sydney (Project No. 2015/336). Informed consent was obtained from all subjects.

### Stimuli

The stimuli were 60 online male profile pictures retrieved from rating and match-making website www.hotornot.com (Fig. 1A for examples). Without transformation, these images were placed on a square canvas (537 pixels in height, subtending 14° of visual angle from the viewing distance of 57 cm). Images varied in composition, face size and background cues. Some images showed men dressed in casual clothes, others were in formal wear or work uniforms. Being profile pictures from a dating website we presume they were intended to attract female attention. All images but one were in colour and three also contained an animal (one dog, one monkey, one dolphin). Images were divided randomly into two sets of 30 (Sets A and B).

For Experiment 2 we used the same 60 profile pictures. For 8 participants, Set A stimuli were presented in their canonical orientation (upright) and Set B stimuli were inverted and for the other 8 participants the order was reversed: Set A inverted and Set B upright. Although some of the face images had pronounced head tilts we did not rotate the faces to horizontally align the eyes (as is common in face perception experiments) because we wanted to preserve ecological validity and our ability to interpret the results in the context of the real world. However, this means that the label “upright” refers to the profile picture as an entire stimulus and not the face *per se* (and likewise for “inverted”). This may be reduced the expected size of the classic face inversion effect.

### Experimental Procedure

Participants completed the experiment in a dimly lit curtained booth. Both experiments were programmed in MATLAB version R2010a using the Psychophysics Toolbox 3^13^,^14^. The program was run on an Apple Mac Pro running Mac OSX Lion 10.7.5. Participants were seated approximately 57 cm in front of a CRT monitor (18 inch viewable screen size) set at a screen resolution of 1024 × 768 pixels with a refresh rate of 100 Hz. Participant responses were recorded using an Apple wired USB keyboard.

In both experiments, a trial began with the presentation of a face for 300 ms. It was replaced with a white fixation cross which remained visible until the participant made a binary decision; was the face attractive or unattractive. Participants used the arrow keys to indicate a decision. As soon as a response was recorded, the next trial began immediately.

In Experiment 1, participants judged 30 faces (each face seen 10 times; 300 trials in total) in a random order. In Experiment 2, participants judged 60 faces (each face seen 10 times; 600 trials in total) but this time 30 faces were upright and remaining 30 faces were rotated 180° in the picture-plane. In both experiments, to minimise local adaptation and predictability, faces were presented in one of eight screen locations equidistant from the fixation cross by 3.5°.

## Additional Information

**How to cite this article**: Taubert, J. *et al.* Love at second sight: Sequential dependence of facial attractiveness in an on-line dating paradigm. *Sci. Rep.* **6**, 22740; doi: 10.1038/srep22740 (2016).
